# Elements of Human Physiology

**Published:** 1893-05

**Authors:** 


ELEMENTS OF HUMAN PHYSIOLOGY, by Ernest H. Starling, M. D., London, M. R. C. P., Joint Lecturer on Physionlogy at Guy's Hospital, London; member of Physiological Society, etc.. Philadelphia. P. Blakiston, Son & Co., 1892. Price, $2.

An excellent little text book that will be welcomed by the student. The subject of Histology has been left out as it more properly belongs to anatomy. All the other subjects usually described in a *text* book of Physiology, are clearly treated and the work is well up to date in all of its departments.

